# Underutilization of Staging Laparoscopy Prior to Neoadjuvant Systemic Therapy in Gastric Cancer

**DOI:** 10.1245/s10434-025-18547-4

**Published:** 2025-10-17

**Authors:** Arsha Ostowari, Kathryn T. Chen, Bima J. Hasjim, Stefania Montero, Shaina Sedighim, Fatemeh Tajik, Melanie Roman, Farshid Dayyani, Shaun Daly, Brian R. Smith, Ninh T. Nguyen, Oliver S. Eng, Michael P. O’Leary, Maheswari Senthil

**Affiliations:** 1 Department of Surgery, University of California, Irvine Medical Center, Orange, CA USA; 2 Division of Surgical Oncology, Department of Surgery, Harbor-UCLA Medical Center, Torrance, CA USA; 3 Department of Surgery, Harbor-UCLA Medical Center, Torrance, CA USA; 4 Division of Hematology/Oncology, Department of Medicine, University of California, Irvine Medical Center, Orange, CA USA; 5 Division of Surgical Oncology, Department of Surgery, University of California, Irvine Medical Center, Orange, CA USA; 6 Chao Comprehensive Cancer Center, University of California, Irvine Medical Center, Orange, CA USA; 7 Division of Gastrointestinal Surgery, Department of Surgery, University of California, Irvine Medical Center, Orange, CA USA; 8 Department of Surgery, Loma Linda University, Loma Linda, CA USA

**Keywords:** Staging laparoscopy, Neoadjuvant systemic therapy, Gastric cancer, Siewert 3 gastroesophageal junction cancer, Peritoneal metastases

## Abstract

**Background:**

Radiographically occult peritoneal carcinomatosis (PC) is a major concern in gastric cancer; hence staging laparoscopy (SL) is recommended prior to initiating treatment, particularly neoadjuvant systemic therapy (NST). However, compliance may vary and could result in understaging. We sought to evaluate the utilization of SL in patients with gastric cancer referred to academic institutions.

**Patients and Methods:**

This is a multi-institution retrospective study of patients with a diagnosis of gastric/gastroesophageal junction (GEJ) Siewert 3 adenocarcinoma who received treatment between 2010 and 2022. Demographics, tumor characteristics, treatment, and recurrence data were collected. Descriptive statistics and multivariate analysis were performed.

**Results:**

A total of 280 patients with gastric/GEJ cancer were identified, of which 75 (26.8%) had clinical stage IV disease and were excluded. Of the remaining 205 patients, 74 (36.1%) underwent upfront surgery and 131 (63.1%) underwent NST. Only 39 (29.8%) patients in the NST group underwent SL, of whom 15(38.4%) were found to have peritoneal metastases; 12 (80%) had gross PC and 3 (20%) had positive cytology. Among patients who underwent surgical resection after NST (*n* = 77), 26 (33.7%) experienced disease recurrence with a median time to recurrence of 11.6 months. The peritoneum (*n* = 10/26, 38.5%) was the most common site of recurrence.

**Conclusions:**

Compliance with SL prior to NST is poor (29.8%), and in the group that underwent SL, 38% of patients were upstaged due to presence of peritoneal metastases. These findings are significant, as the management and prognosis of peritoneal metastases are drastically different. Various factors could lead to poor compliance with SL, hence better compliance and alternate approaches to reliably detect PC are needed.

Gastric cancer is the fifth most common cancer and fifth leading cause of cancer deaths worldwide in 2022.^1^ In the USA, in 2025, there are an estimated 30,300 new gastric cancer cases and 10,780 gastric cancer deaths expected to occur.^2^ The age-adjusted rates of new gastric cancer cases have been increasing by an average of 0.4% each year from 2013 to 2022 and around 36% of patients have distant metastatic disease at time of diagnosis. Unfortunately, the 5-year survival rate for patients with metastatic disease is extremely poor, at 7.5%, compared with 76.5% for localized disease and 37.2% for regional disease.^2^ The high incidence of distant metastasis seen at presentation is attributed to multiple factors, such as aggressive biology, race/ethnic differences, delay in diagnosis, and barriers to access to care.^3^ Nevertheless, it portends an extremely grim prognosis. Hence, timely identification and appropriate management of metastatic disease is crucial to improve patient outcomes.

The peritoneum is a common site of metastasis in gastric cancer both at the time of initial presentation and subsequently.^3–5^ Radiographically occult peritoneal metastasis occurs in about 30–40% of patients with newly diagnosed gastric cancer.^3,6,7^ In about 6–11% of these patients, this includes microscopic presence of free cancer cells in the peritoneal lavage fluid, i.e., cytology positive disease (CYT+).^8,9^ The survival outcomes for CYT+ disease are similar to that of patients with macroscopic peritoneal metastasis and therefore classified as stage IV disease. Although the sensitivity of peritoneal lavage cytology is poor, the specificity is high.^10^ Hence, staging laparoscopy (SL) with peritoneal lavage is recommended as part of the staging work up for gastric cancer according to the National Comprehensive Cancer Network (NCCN) guidelines.^11^ Since management of metastatic gastric cancer, both in terms of systemic therapy and surgical approach, is different than locoregional resectable gastric cancer, appropriate staging prior to initiating neoadjuvant systemic therapy (NST) is critical to avoid understaging and undertreating patients. However, compliance with SL prior to initiation of NST may be variable across different practice settings. We sought to evaluate the use of SL prior to NST among patients referred to two large academic institutions.

The primary outcome of our analysis was the utilization of SL prior to initiation of NST in patients with gastric and Siewert type 3 GEJ adenocarcinoma. Secondary outcomes included the proportion of patients upstaged after SL due to the presence of PC and the recurrence patterns after NST.

## Patients and Methods

We conducted a multi-institutional retrospective study of patients with a new diagnosis of gastric/GEJ cancer between 1 January 2010 and 31 December 2022. Patients eligible for the study were ≥ 18 years old, had a new diagnosis of adenocarcinoma of gastric or gastroesophageal origin that was classified as Siewert type 3, and received all or some of their treatment at either the University of California, Irvine Medical Center, or Harbor-UCLA Medical Center.

A comprehensive review of all patients’ electronic medical records was performed and data pertaining to demographics (age, sex, race/ethnicity, insurance type), date of diagnosis, tumor characteristics (location, histology, grade, and clinical stage), receipt of SL, treatment (NST, type of surgical intervention), recurrence and survival were collected. Gastric/GEJ cancers were identified on computed tomography (CT) imaging or esophagogastroduodenoscopy (EGD) and confirmed by endoscopic biopsy. Endoscopy reports were reviewed to ascertain the Siewert classification and only Siewert type 3 GEJ adenocarcinomas (located 2–5 cm distal to the gastric cardia that infiltrates the GEJ and esophagus from below^1,2^) were included in this study.

The patient cohort was stratified on the basis of the presence or absence of SL prior to initiation of NST. SL was defined as a laparoscopy performed after the diagnosis of a gastric or GEJ cancer but prior to the initiation of NST with the intention to rule out the presence of gross or microscopic PC. Descriptive statistics were used to analyze demographic and clinicopathologic covariates. Means and medians were reported with their interquartile range (IQR), respectively. Logistic regression analysis of factors associated with performance of staging laparoscopy was performed.

## Results

### Patient/Tumor Characteristics

**Fig. 1:**
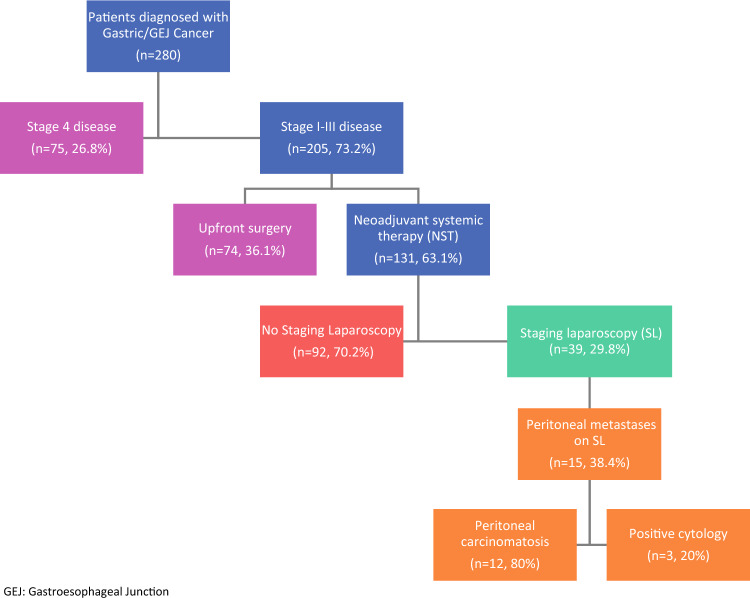
Flow chart of total patient cohort with gastric/gastroesophageal junction (GEJ) cancer

A total of 280 patients with gastric/GEJ cancer were identified during the study period, of whom 75 (26.8%) had clinical stage IV disease on the basis of distant metastases seen on imaging at the time of diagnosis and were excluded (Fig. 1). Of the remaining 205 (73.2%) patients with clinical stage I–III disease, 74 (36.1%) underwent upfront surgical intervention and 131 (63.1%) underwent NST as the first course of treatment. Among the patients who underwent NST, only 39/131 (29.8%) underwent SL prior to NST to evaluate for peritoneal metastases, and of the remaining 92 patients, 31 (33.7%) had SL after initiation of NST, but prior to definitive surgical resection. Collectively, 70/131 (53.4%) patients in the NST group had a staging laparoscopy, of whom 41/70 (58.5%) had peritoneal washings and cytology performed. Of the patients who did not undergo SL prior to NST, 26/92 (28.3%) had chemotherapy initiated at an outside practice prior to referral to a tertiary hospital and evaluation by a surgeon.

The demographic information of patients who received NST, grouped by receipt of laparoscopy [SL or no SL (NSL)] are presented in Table 1. The median follow-up period was 30.6 months. The median time to SL following diagnosis was 17 days. The majority of patients in both groups were male (SL 66.7%; NSL 63.0%, *p* = 0.844), Hispanic race (SL 53.9%; NSL 41.3%, *p* = 0.696), and had government-funded insurance (SL 61.5%; NSL 72.5%, *p* = 0.317). In terms of tumor characteristics presented in Table 1, the majority were gastric cancers (SL 82.1%; NSL 79.3%, *p* = 0.625) and poorly differentiated (SL 84.6%; NSL 79.6%, *p* = 0.630). Presence of signet ring cell component was observed in 52.6% of SL and 50.6% of NSL group (*p* = 0.943). Patients who underwent SL more often had a higher clinical stage (stage III 59.0% versus 33.7%, *p* = 0.015) and were younger compared with the NSL group (median age of 59 versus 62 years, *p* = 0.011).

**Table 1:** Demographic and tumor characteristics of patients who underwent neoadjuvant systemic therapy grouped by receipt of staging laparoscopy

Characteristics	Staging laparoscopy (SL)	No staging laparoscopy (NSL)	*p*-Value
*N (%)*	39 (29.8%)	92 (70.2%)	
*Median age (years) (IQR)*	59 (47.5–64)	62 (55–70.25)	0.011
*Sex (n, %)*			0.844
Male	26 (66.7%)	58 (63%)	
Female	13 (33.3%)	34 (37%)	
*Race/ethnicity (n, %)*			0.696
Non-Hispanic white	9 (23.1%)	23 (25.0%)	
Asian	7 (18.0%)	26 (28.3%)	
Hispanic	21 (53.9%)	38 (41.3%)	
Non-Hispanic Black	1 (2.6%)	3 (3.3%)	
Mixed	1 (2.6%)	2 (2.2%)	
*Insurance (n, %)*			0.317
Government	24 (61.5%)	66 (72.5%)	
Private	7 (18.0%)	15 (16.5%)	
None	8 (20.5%)	10 (11.0%)	
Unknown	0 (0)	1 (1.1%)	
*Diagnosis (n, %)*			0.625
Gastric cancer	32 (82.1%)	73 (79.3%)	
GEJ cancer	7 (17.9%)	19 (20.7%)	
*Tumor Location (n, %)*			0.053
Proximal	5 (12.8%)	26 (28.3%)	
Body	7 (18.0%)	29 (31.5%)	
Distal	15 (38.5%)	21 (22.8%)	
Overlapping	8 (20.5%)	11 (12.0%)	
NOS	4 (10.3%)	5 (5.4%)	
*Histology (n, %)*			0.943
Intestinal	8 (21.0%)	14 (17.7%)	
Diffuse	9 (23.7%)	21 (26.6%)	
Signet	20 (52.6%)	40 (50.6%)	
Papillary	1 (2.6%)	2 (2.5%)	
Tubular	0 (0)	1 (1.3%)	
NOS	1 (2.6%)	14 (15.2%)	
Grade (*n*, %)			0.630
Well differentiated	3 (7.7%)	6 (6.8%)	
Moderately differentiated	3 (7.7%)	12 (13.6%)	
Poor/undifferentiated	33 (84.6%)	70 (79.6%)	
NOS	0 (0)	4 (4.3%)	
*Clinical stage, AJCC (n, %)*			0.015
I	4 (10.3%)	13 (14.1%)	
II	12 (30.8%)	35 (38.0%)	
III	23 (59.0%)	31 (33.7%)	
NOS	0 (0)	13 (14.1%)	

^*^Government insurance: consists of government sponsored insurance programs such as Medi-Cal and Medicare

*NOS* not otherwise stated, *GEJ* gastroesophageal junction

Of the 39 patients who underwent SL prior to the initiation of NST, 15 (38.4%) were upstaged to stage 4 disease due to the presence of peritoneal metastases; 12/39 (30.7%) patients had gross PC; and 3/39 (7.7%) patients had CYT+ disease. Among the patients who underwent SL after initiation of NST, 5/31 (16.1%) were found to have peritoneal disease (4 CYT+ and 1 PC). On logistic regression multivariable analysis, age was the only significant factor associated with performance of staging laparoscopy (OR 0.95, 95% CI 0.91–0.99, *p* = 0.01) (Table 2).

**Table 2:** Logistic regression analysis of factors associated with performance of staging laparoscopy

Characteristics	Univariate		Multivariate	
	OR (95% CI)	*p*-Value	OR (95% CI)	*p*-Value
*Age*	0.95 (0.92–0.98)	< 0.001	0.95 (0.91–0.99)	0.01
*Race*				
Non-Hispanic white	Reference		Reference	
Non-Hispanic Black	0.59 (0.03–5.45)	0.670	0.19 (0.00–3.56)	0.330
Hispanic	1.13 (0.43–3.11)	0.810	1.56 (0.45–5.83)	0.490
Asian	0.50 (0.15–1.59)	0.240	0.65 (0.17–2.54)	0.530
Mixed	0.89 (0.04–10.60)	0.930	2.14 (0.07–68.74)	0.630
*Insurance*				
Private	Reference		Reference	
Uninsured	2.29 (0.57–9.82)	0.250	2.86 (0.57–15.89)	0.210
Government	0.69 (0.24–2.03)	0.480	0.62 (0.19–2.12)	0.430
*Grade*				
Well	Reference		Reference	
Moderate	0.55 (3.76–0.53)	0.530	0.46 (0.06–3.65)	0.450
Poor	1.12 (0.28–5.57)	0.880	0.99 (0.21–5.61)	0.990
*Clinical stage, AJCC*				
I	Reference		Reference	
II	1.11 (0.32–4.54)	0.870	1.12 (0.27–5.29)	0.880
III	2.41 (0.74–9.44)	0.170	2.28 (0.60–10.34)	0.250

The type of surgical resection after NST is presented in Table 3. The most common surgical procedure in both groups was a distal subtotal gastrectomy (SL 43.5% and NSL 35.9%) followed by total gastrectomy (SL 21.7% and NSL 17.4%). No definitive surgical intervention was performed in 5/23 (21.7%) of SL and 29/92 (31.5%) of NSL patients. The most common reason for not proceeding with definitive surgical resection was disease progression. Time to recurrence and sites of recurrence for patients with stage I–III who underwent curative resection are presented in Table 4. The median time to recurrence was 9.8 and 11.6 months for the SL and NSL group, respectively (*p* = 0.846). The recurrence rate was 44.4% in the SL and 30.5% in the NSL group (*p* = 0.418). The peritoneum was the most common site of metastases in both groups (SL 62.5 % and NSL 27.8%, *p* = 0.519) (Table 4).

**Table 3:** Surgical intervention after neoadjuvant systemic therapy for patients with clinical stage I–III disease grouped by staging laparoscopy

Characteristics	Staging laparoscopy	No staging laparoscopy
*N (%)*	23 (20%)	92 (80%)
No surgery	5 (21.7%)	29 (31.5%)
*Type of definitive surgery*		
Partial gastrectomy	10 (43.5%)	33 (35.9%)
Total gastrectomy	5 (21.7%)	16 (17.4%)
Esophagogastrectomy	3 (13%)	10 (10.9%)
Surgery aborted	0	4 (4.3%)

*One patient excluded from staging laparoscopy cohort because of development of stage IV disease requiring CRS

**Table 4:** Recurrence data for patients with clinical stage I–III disease who underwent surgical treatment after neoadjuvant systemic therapy grouped by staging laparoscopy

Characteristics	Staging laparoscopy	No staging laparoscopy	Total cohort	*p*-Value
*N* (%)	18 (23.4%)	59 (76.6%)	77 (100%)	
Number of patients with recurrence	8 (44.4%)	18 (30.5%)	26 (33.7%)	0.418
Median time to recurrence	9.8 months	11.6 months	11.6 months	0.846
Sites of recurrence*				0.519
Lymph nodes	2 (25.0%)	3 (16.7%)	5 (19.2%)	
Peritoneum	5 (62.5%)	5 (27.8%)	10 (38.5%)	
Liver	0 (0)	3 (16.7%)	3 (11.5%)	
Lung	0 (0)	4 (22.2%)	4 (15.4%)	
Ovary	0 (0)	1 (5.6%)	1 (3.8%)	
Meninges	0 (0)	1 (5.6%)	1 (3.8%)	
Locoregional	1 (12.5%)	3 (16.7%)	4 (15.4%)	

*Some patients experienced multiple sites of recurrence

## Discussion

Although the likelihood of radiographically occult peritoneal metastasis in gastric cancer has been well recognized, compliance with SL prior to NST in this study was only around 30%. The significance of this finding is further augmented by the observation that 38.4% of patients who underwent SL had peritoneal metastasis and were upstaged to stage IV disease. Besides the significant difference in 5-year survival rates between distant disease and locoregional disease, the management of patients with stage IV disease has also significantly evolved over the past 5 years.^2^ Recent randomized controlled trials in metastatic gastric cancer have shown that the combination of targeted therapies such as trastuzumab, zolbetuximab, and/or immune checkpoint inhibitors and systemic therapy are associated with improved survival compared with systemic chemotherapy alone in the first-line setting.^12–15^ Hence, understaging of patients would deprive them of the opportunity to be appropriately treated for metastatic disease with these targeted therapies and will unequivocally result in poorer outcomes.

Consistent with previously published data, the incidence of peritoneal metastasis in the SL group was 38.4%.^6,8,16^ This leads to the obvious concern that a similar percentage of patients in the NSL group may have had peritoneal metastasis that was undetected prior to NST. This concern is further validated by the finding that 16% of patients were found to have peritoneal disease during subsequent SL, and overall, 31.5% of patients in the NSL group did not receive a curative surgical intervention after NST often due to disease progression.

The higher rate of recurrence in the SL group could likely be attributed to the higher percentage of patients with stage III disease in this group compared with the NSL group (59.0% versus 33.7%, *p* = 0.015). Additionally, about 31.5% in the NSL did not undergo surgical resection due to disease progression and should be considered while assessing the recurrence rates between the two groups. The median time to recurrence of under 1 year in both groups in this study is likely explained by the tumor characteristics seen in the patient cohorts. More than 75% of patients in both groups had poorly differentiated carcinoma and more than 50% had signet ring cells present. Similarly, the peritoneum being the most common site of metastasis is also supported by the tumor biology, as poorly differentiated and signet ring histologies have a higher predilection to metastasize to the peritoneum.^17^

The reason for poor compliance with SL in this study is likely multifactorial. Due to the retrospective nature of the study, the exact reason for omission of SL in every patient could not be accurately elucidated. However, the observation that 28% of patients had NST started at outside practice prior to referral to a tertiary center indicates practice preferences of medical oncologists in community settings. Other factors such as insurance barriers, institutional practices, and urgency to start systemic treatment may have contributed to poor compliance with SL. Moreover, lack of adherence to a standardized approach of SL and omission of peritoneal washing/cytology further amplify the issue.

There are several limitations to our study. First, due to the retrospective nature of the study, the exact reasons that led to omission of SL were not available. Second, the exact operative details of SL were also not available in many cases. The performance of cytology was largely determined by the associated pathology/cytology results. Third, the findings of this study are from two academic institutions; hence, the results may not be generalizable. There may be practice settings in which SL is performed routinely with high rate of compliance. Nevertheless, we believe our findings warrant reporting to create awareness about the risk of missing peritoneal metastases in nearly 40% of patients with GC when SL is omitted. Additionally, there may be an opportunity to standardize SL technique and reporting and increase the rate of performance of peritoneal washings in institutions that routinely care for patients with gastric cancer.

Despite the multitude of reasons behind poor compliance with SL, under diagnosing the deadliest form of metastasis, i.e., peritoneal metastasis, is a major issue. This is particularly relevant as the management of metastatic gastric cancer limited to the peritoneum is evolving, with increased emphasis on the role of intraperitoneal chemotherapy in combination with systemic therapy to improve patient outcomes. The results of the DRAGON-01 phase III clinical trial from China demonstrated a statistically significant increase in overall survival with normothermic iterative intraperitoneal paclitaxel combined with intravenous (IV) paclitaxel and oral S-1 (19.4 versus 13. 9 months, *p* < 0.0001) compared with IV paclitaxel and oral S-1 in patients with gastric carcinomatosis.^18^ In the USA, EA2234 – STOPGAP II trial, a national randomized phase II/III clinical trial, is about to launch and will investigate the role of bidirectional paclitaxel combined with systemic therapy versus systemic therapy alone in patients with gastric/GEJ Siewert 3 adenocarcinoma with synchronous peritoneal disease (cyt+ or peritoneal carcinomatosis) (NCT07001748). This study is a follow-up to the phase II single-institution STOPGAP I trial (NCT04762953).^19^ In addition to normothermic intraperitoneal chemotherapy (NIPEC), there are clinical trials with heated intraperitoneal chemotherapy (HIPEC) (NCT 03348150, 04308837,05753306, not an exhaustive list) and pressurized intraperitoneal aerosolized chemotherapy (PIPAC) (NCT04329494, 05644249, 05303714, not an exhaustive list) for the treatment of gastric carcinomatosis. Hence, underdiagnosing patients with peritoneal disease not only limits the type of systemic treatment, but may also deprive patients of the opportunity to participate in clinical trials specifically designed to address peritoneal metastases.

While efforts to improve multidisciplinary care with involvement of surgeons from the beginning of cancer care continuum through community outreach and education are important, an additional strategy would be to develop novel approaches to diagnose peritoneal disease using routine staging computed tomography scans with artificial intelligence deep machine learning models. Our team has developed such a model and is currently in the process of validating it in a large international cohort of patients.

## Conclusions

The compliance with SL prior to initiation of neoadjuvant systemic therapy to rule out radiographically occult peritoneal metastasis is poor. Additionally, the incidence of peritoneal metastasis in patients in our cohort who undergo SL is 38%. Improvements in the management of patients with metastatic gastric cancer call for efforts to increase compliance with SL through education, reducing barriers to care and improving multidisciplinary approach. In addition, there is also an opportunity to develop artificial-intelligence-based diagnostic tools to identify or predict the risk of peritoneal disease on the basis of routine imaging studies and clinical risk factors.
